# Complexity and Controversies over the Cytokine Profiles of T Helper Cell Subpopulations in Tuberculosis

**DOI:** 10.1155/2015/639107

**Published:** 2015-10-01

**Authors:** Marcos Vinicius da Silva, Monique Gomes Salles Tiburcio, Juliana Reis Machado, Djalma Alexandre Alves Silva, Denise Bertulucci Rocha Rodrigues, Virmondes Rodrigues, Carlo Jose Freire Oliveira

**Affiliations:** ^1^Laboratory of Immunology, Federal University of Triangulo Mineiro, 38025-180 Uberaba, MG, Brazil; ^2^Department of Pathology, Institute of Tropical Pathology and Public Health, Federal University of Goias, 74605-050 Goiania, GO, Brazil; ^3^Cefores, Federal University of Triangulo Mineiro, 38015-050 Uberaba, MG, Brazil; ^4^Laboratory of Biopathology and Molecular Biology, University of Uberaba, 38055-500 Uberaba, MG, Brazil

## Abstract

Tuberculosis (TB) is a contagious infectious disease caused by the TB-causing bacillus *Mycobacterium tuberculosis* and is considered a public health problem with enormous social impact. Disease progression is determined mainly by the balance between the microorganism and the host defense systems. Although the immune system controls the infection, this control does not necessarily lead to sterilization. Over recent decades, the patterns of CD4+ T cell responses have been studied with a goal of complete understanding of the immunological mechanisms involved in the maintenance of latent or active tuberculosis infection and of the clinical cure after treatment. Conflicting results have been suggested over the years, particularly in studies comparing experimental models and human disease. In recent years, in addition to Th1, Th2, and Th17 profiles, new standards of cellular immune responses, such as Th9, Th22, and IFN-*γ*-IL-10 double-producing Th cells, discussed here, have also been described. Additionally, many new roles and cellular sources have been described for IL-10, demonstrating a critical role for this cytokine as regulatory, rather than merely pathogenic cytokine, involved in the establishment of chronic latent infection, in the clinical cure after treatment and in keeping antibacillary effector mechanisms active to prevent immune-mediated damage.

## 1. Introduction

Tuberculosis (TB) is a contagious infectious disease caused by the TB-causing bacillus* Mycobacterium tuberculosis* and is considered a public health problem with enormous social impact. Approximately 8.6 million new TB cases and 1.6 million deaths are recorded annually; therefore, this illness is a major cause of death worldwide [1]. Transmission of* M. tuberculosis* occurs by inhalation of droplets containing these bacilli that are eliminated in the sputum of an individual with active disease. In most cases, approximately 90–95%,* M. tuberculosis* infection is clinically asymptomatic and not transmitted, a state referred to as latent tuberculosis. It is estimated that one-third of the world population is infected with* M. tuberculosis*, but only 5–10% will develop active disease at some point in their lives [1, 2].

Disease progression is determined mainly by the balance between the microorganism and host defense systems and major changes in the immune status of the individual potentiate TB activation or reactivation [3]. Although the immune system controls the infection, this control does not necessarily lead to sterilization. Once* M. tuberculosis* can be found in vacuoles of macrophages, the protective immune response against mycobacteria is dependent on the interaction between these host cells and CD4+ T cells. Depletion of CD4 or MHC class II molecules in mice impairs control of bacterial growth, and animals succumb to the disease [4, 5]. Similarly, HIV patients with reduced CD4+ T cells are highly susceptible to tuberculosis [6].

Over recent decades, the patterns of CD4+ T cell responses have been studied, with the goal of complete understanding of the immunological mechanisms involved in the maintenance of latent or active tuberculosis infection and of the clinical cure after treatment. Conflicting results have been suggested over the years, particularly in studies comparing experimental models and human disease. In recent years, in addition to Th1, Th2, and Th17 profiles, new standards of cellular immune response, such as Th9 and Th22, have also been described (Table 1). Similarly, several studies have pointed IL-10 as a crucial regulator to determine the quality and intensity of the immune response, demonstrating a major role in the establishment of latent infection, to prevent immune-mediated damage and establishment of clinical cure after treatment keeping the antibacillary effector mechanisms.

## 2. T Helper Cells and Immune Response in Tuberculosis

T cell-mediated immune response begins after dissemination of* M. tuberculosis* to the lymph nodes [66, 67]. After activation and expansion of antigen-specific T cells, they then migrate to the infected lungs where they are found, together with other leukocytes, as part of granulomas. Several distinct types of T helper cells (such as Th1, Th2, Th17, and regulatory T cells) are present at the site of infection (Table 1); however, the Th1 subset is classically associated with impaired growth and dispersion of* Mycobacterium tuberculosis* [68]. Because each pattern of immune response culminates in different effector mechanisms, it is essential to understand the role of each one in response to* M. tuberculosis*.

### 2.1. Th1

IFN-*γ*, the main cytokine of the Th1 profile, enhances macrophage microbicidal mechanisms because it activates signaling pathways that include the iNOS pathway [39, 40] and induces the process of acidification and maturation of phagosomes and autophagy [41]. The main source of the IFN-*γ* that is responsible for the control of* M. tuberculosis* is CD4+ T cells [69]. Additional roles in the production of that cytokine are attributed to CD8+ T cells, natural killer cells, *γδ* T cells, and CD-1 restricted T cells; however, none of them can compensate for the absence of CD4+ T cells [68]. The importance of IFN-*γ* in response to* M. tuberculosis* has been widely investigated in experimental models and in humans. Knockout mice for IL-12 [7], IFN-*γ* [8, 9], or T-bet [10] are highly susceptible to TB. It was also demonstrated that a reduction in IFN-*γ* may lead to an increased influx of neutrophils and extensive tissue damage resulting in tuberculosis in animal models [38]. Individuals with mutations in the IL-12/IFN-*γ* axis develop disseminated infection caused by BCG or nontuberculous species of mycobacteria [11]. Furthermore, results from our group and other studies have also demonstrated that peripheral blood mononuclear cells (PBMCs) from patients with active disease secrete lower levels of IFN-*γ in vitro*, both in unstimulated cultures or after stimulation with mitogens or mycobacterial antigens [13, 33, 58, 60, 70–74]. A recovery of the ability to produce IFN-*γ* after specific antituberculosis therapy was also demonstrated, but this production was found at low levels when compared with patients with latent tuberculosis [74, 75].

Despite the important role of IFN-*γ* in the fight against* M. tuberculosis*, some studies have shown that production of this cytokine is not sufficient to prevent active disease. Most people who develop active TB are able to activate IFN-producing T cells that are specific for* M. tuberculosis* at the site of infection [34–37]. It has been shown that patients whose T cells produce higher amounts of IFN-*γ* are more likely to progress to active disease than patients with weaker responses [76]. One possible explanation for the contradictory results regarding the production of IFN-*γ* at the site of infection and in peripheral blood is that the PBMCs of patients with active disease are more susceptible to apoptosis than healthy controls [77]. Moreover, in patients with active disease, T cells specific for mycobacterial antigens have been shown to be recruited and retained in lung tissues. In fact, several authors have shown that there is sometimes a positive relationship between circulating T-cell clones and those retained in the site of infection, especially in patients with active disease [78–80].

The notion that IFN-*γ* is necessary but not sufficient for bacterial control after infection is also supported by several studies in knockout mice for TNF-*α*, granulocytes, GM-CSF, IL-1, and IL-6, as they also die rapidly after* M. tuberculosis* infection. In other words, these results suggest that additional pathways are essential for immunity against* M. tuberculosis* [81]. Several experimental models have also linked the production of TNF-*α* with the maintenance of granuloma integrity, and changes in their levels have been correlated with disease susceptibility both in experimental models and in human patients [33, 42–44]. In fact, TNF-*α* acts synergistically with IFN-*γ* to stimulate the production of NO by macrophages and influences the expression of chemokines, such as CCL5, CCL9, CXCL10, and CCL2, which induce migration to and maintenance of immune cells in the infection site [82]. Blocking TNF-*α*, for example, in the treatment of rheumatoid arthritis, leads to a loss of granuloma structure and reactivation of the disease [45–48]. Conversely,* M. tuberculosis*-specific stimulation of IFN-*γ* (but not TNF-*α*) and IFN-*γ*R signaling are significantly depressed in active TB, which correlates with TB disease severity and activity. Thus, the depression of both TNF-*α* and IFN-*γ* production and IFN-*γ*R signaling may synergize to contribute to defective host control in active TB [74].

### 2.2. Th2

The role of Th2 cytokines, classical antagonists of the Th1 profile, has not been fully elucidated in experimental models or in patients with tuberculosis. Although these cytokines may be involved in mechanisms of evasion of* M. tuberculosis* from the immune system, their direct participation in disease reactivation is even more controversial [83]. In experimental models, some studies have shown an association of increased IL-4 with progression of tuberculosis and reactivation of the disease [15, 54], but other authors have shown that the absence of this cytokine does not influence susceptibility to the disease [55, 56].

In human tuberculosis controversial results are also observed with respect to induction of the Th2 subset of CD4+ T cells. Some authors have demonstrated increased levels of IL-4 in the blood [49–52] and in bronchoalveolar lavage fluid (BALF) in patients with TB, especially those with the more severe forms [53]. Ashenafi et al. have recently demonstrated an association between high levels of IL-4 and CCL4 in BALF, increased expression of SOCS3, and clinical progression of the disease. Furthermore, a positive correlation was found between IL-4 and CCL4 levels and mycobacteria-specific IgG titers in BALF and plasma samples [12]. In other studies, it was observed that the decrease in Th1 response in patients with active disease is not accompanied by an increase in the Th2 pattern [13, 14]. When examining the kinetics of Th1 and Th2 cytokine production in patients with active disease, the secretion of both types of cytokines has been shown by using PBMCs from active TB patients [84]. Also, Marchant and colleagues demonstrated that, prior to therapeutic intervention, there is a predominance of “Th0” cells because concomitant expression of IFN-*γ* and IL-4 was found; however, with treatment progression, there was a decrease in clones producing IL-4. In contrast, patients with treatment-resistant tuberculosis showed an increase in “Th0” cells because there was persistent production of IL-4 regardless of the presence or absence of treatment [84].

The participation of Th2 cytokines in susceptibility to tuberculosis was reinforced by the description of the importance of autophagy in immunity against* M. tuberculosis* [41, 85]. It is known that IL-4 and IL-13 cytokines act as inhibitors of the autophagic process [57, 86], impairing antigen presentation, T cell clonal expansion, and consequently the organization of granulomas in tuberculosis [85–87]. However, it remains unclear whether the production of Th2 cytokines is a primary cause of reactivation of tuberculosis or just a consequence of progression of the active infection [83, 88].

### 2.3. T Regulatory

In many chronic infections, including TB, immune-mediated tissue injury can lead to greater damage to the host when compared with the presence of the pathogen itself. Thus, it is important that mechanisms are activated to counterregulate proinflammatory immune responses and prevent the harmful effects of excessive inflammation [81]. Regulatory T cells (Foxp3+ CD4+ and CD25+ (Treg)) suppress inflammation and limit immune responses by producing immunosuppressive cytokines, such as IL-10, IL-35, and TGF-*β*, and directly interacting with other cells by inhibiting surface molecules [89].

The role of Treg cells in tuberculosis, especially in human disease, still remains controversial. Some studies suggest that the involvement of Treg cells is observed more in individuals with more severe active disease [16], and depletion of these cells results in the production of high levels of IFN-*γ* [33, 90–93]. Previous results also suggest an important role for regulatory T cells in reactivation of latent tuberculosis and development of active tuberculosis by decreasing the production of IFN-*γ*, while production of IL-17 continues to induce the accumulation of polymorphonuclear leukocytes at lung sites [17]. Additionally, one study suggests that a concomitant increase in the proportion of CD4+ CD25+ and CD4+ CD25+ Foxp3+ cells are associated with active pulmonary tuberculosis [94], but the depletion of CD4+ CD25+ cells has no effect on bacterial load or lung damage due to infection [95]. A recent study by Chen and colleagues showed that, in experimental tuberculosis, concomitant expansion of regulatory T cells (Foxp3+ T cells) and effector T cells is already occurring in the early days of infection, and this increase is critical for control of the parasite because it protects from severe disease [19]. Despite these results, it is known that the persistent stimulation in multidrug-resistant tuberculosis increases Treg cells and could be a cofactor in clinical cure delay [18].

### 2.4. Th17

Activation of naïve T cells in the presence of TGF-*β* and IL-6 directs the differentiation of these cells into Th17 cells through activation of STAT-3. This in turn increases expression of the transcription factor ROR*γ*t and promotes the production of both proinflammatory cytokines IL-17 and IL-22 [96]. The induction of IL-21 mediated by IL-6 also strengthens the engagement of the Th17 strain in an autocrine fashion. It is known that TGF-*β* is produced in excess during tuberculosis and is expressed at sites of active* M. tuberculosis *infection [97], suggesting their involvement in the differentiation of Th17 cells in addition to its well-known immunomodulatory role. For tuberculosis, it has been shown that IL-23 is required for the development of Th17 cells because mice deficient in the p19 subunit of IL-23 are unable to maintain sustained expression of IL-17 during the course of infection [98]. In the same study, however, it was suggested that the Th17 response is dispensable for protection against infection.

Wozniak et al. suggest that cross-regulation of Th1 and Th17 populations is essential for conferring a significant protective effect against* M. tuberculosis* without excessive damage [25]. In agreement with these findings, another study showed that, during infection with* M. tuberculosis*, IFN-*γ* inhibits the production of IL-17 by CD4+ T cells, impairing the survival of neutrophils and the accumulation of these cells in infected lungs, which contributes to a reduction in inflammation [38]. Cruz et al. showed that IFN-*γ*-deficient mice infected with mycobacteria exhibit intensified accumulation of neutrophils and IL-17-producing T cells in the granulomatous lesions and that these cells did not control the growth of bacteria and yet compromised the integrity of the infected tissue [22]. These data suggest that IFN-*γ* appears to limit the population IL-17-producing cells. The involvement of IL-17 and IL-23 in mediating the immunopathology of TB has also been demonstrated by Cruz et al. [23].

In contrast, some studies have indicated that cells producing IL-17 may confer protection in patients with tuberculosis. Khader et al. demonstrated in an experimental model that IL-17 is required to accelerate the recruitment of cells producing IFN-*γ* in the lung, and this effect is a result of increasing concentrations of the chemokines CXCL9, CXCL10, and CXCL11 [24]. Recently, Gopal et al. suggested that an IL-17 mediated response is required for induction of a Th1 response following vaccination with BCG [62]. Other studies have also shown that Th17 cells, in addition to inducing the early events of granuloma formation [26] and remaining as long-lived memory cells [25, 26, 99], can also mediate mechanisms of protection independent of IFN-*γ* [25, 27]. In humans, some studies have noted that there is a deficient Th17 response in patients with active TB, especially when compared with latent TB, a finding that does not seem to be related to a large recruitment of these cells to the lung environment [20, 21].

### 2.5. Th22

IL-22 is a member of the IL-10 family of cytokines, which are mainly produced by Th17 cells. However, recently, a subpopulation of human T cells producing IL-22 has been described as a separate helper T cell line known as Th22. The differentiation of these cells occurs from naïve precursors and is dependent on IL-6 and TNF-*α* via activation of the transcription factor aryl-hydrocarbon receptor (AhR) [100]. It appears that IL-22 is important for inflammatory responses in the skin and mucosal surfaces because it has been reported in a number of human diseases, including inflammatory bowel disease, psoriasis, and rheumatoid arthritis [101]. Despite reports that IL-22 deficiency or neutralization does not alter the outcome of* M. tuberculosis* infection in mice [32, 65], studies in patients with tuberculosis have shown the presence of IL-22-producing CD4+ T cells: Scriba et al. demonstrated that a substantial proportion of mycobacteria-specific Th cells from healthy* M. tuberculosis*-exposed individuals produce IL-22 and are distinct from Th17 and Th1 cells, implicating IL-22 as an important cytokine axis in human antimycobacterial immunity [20]. Similarly, Qiu et al. demonstrated that there is an intense production of IL-22 and IFN-*γ* by distinct subsets of CD4+ T cells in cultures of PBMCs from patients with active TB, and these populations were reciprocally regulated after blocking such cytokines in culture with monoclonal antibodies [28].

Patients with tuberculous pleural effusion have an increased concentration of IL-22 and Th22 cells in pleural fluid samples that exceed the corresponding blood levels in the same patients, suggesting that this cytokine may be involved in pathogenesis of the disease [29–31]. Most Th22 cells in the pleural effusion exhibited a phenotype of effector memory cells, expressing high levels of CD45RO and low levels of CD45RA and CD62L. In addition, it was shown that IL-1*β*, IL-6, and TNF-*α* can promote the differentiation of Th22 cells from naïve CD4+ T cells and that combinations of these cytokines promote differentiation at more pronounced levels [63].

### 2.6. Th9

IL-9 has long been considered a Th2 cytokine due to its participation in processes of allergic inflammation. However, recent studies have revealed that this cytokine has other important functions and distinct subpopulations of CD4+ T cells, called Th9, are able to produce them. Th9 cells are characterized by production of IL-9 and IL-10 and develop from a naïve CD4+ precursor in the presence of TGF-*β* and IL-4 [102]. Some studies show that Th9 cells can trigger inflammation and contribute to the development of allergic diseases [64]; however, the roles of these cells in infectious diseases, including tuberculosis, are not well established.

Recently, Ye and collaborators demonstrated the presence of Th9 cells in patients with tuberculous pleural effusion. The differentiation of Th9 cells from CD4+ cells that were isolated from the pleural effusion or blood of these patients was dependent on TGF-*β*, and the production of IL-9 in the cultures was amplified by the addition of IL-4, IL-1*β*, and IL-6. It was also suggested in the same work that IL-9 may be, along with TGF-*β*, promoting the differentiation of Th17 cells because a positive correlation was observed between the number of Th9 and Th17 cells in the pleural effusion [63].

Although the participation of Th9 cells in tuberculosis has not been reported in other studies, production of the cytokine IL-9 has been widely demonstrated in some studies. One example is that patients with pulmonary TB had significantly higher levels of IL-6 and IL-9 compared to healthy controls [103, 104]. Hur et al. found that IL-9 production, together with IL-5, IL-13, and IL-17 in response to antigens ESAT-6/CFP-10, can potentially differentiate between latent* M. tuberculosis* infection and infections with environmental mycobacteria, such as* M. avium* and* M. kansasii* [105]. Moreover, in patients with latent TB, the addition of exogenous IL-9 reduced the expression of IFN-*γ* by PBMCs* in vitro*, and neutralization of IL-9 restored IFN-*γ* production, suggesting that IL-9 may contribute to the development of TB by promoting an impaired Th1 response [106].

## 3. IL-10 and Tuberculosis—A Delicate Balance between Bacillary Persistence and Reducing Damage 

Several studies have indicated that the recurrence of tuberculosis is associated more with reemergence of a previous infection than with a new infection, reinforcing the concept that antituberculosis immunity that is generated after treatment culminates in clinical recovery but without resulting in a sterilizing cure. This aspect seems crucial because immune responses mediated by both effector (especially Th1 and Th17) and regulatory (T regulatory cells and IL-10) mechanisms are required to allow the patient to fight against the bacilli without suffering extensive lung damage or death. Indeed, because the activity of unrestrained TNF and IFN-*γ* can be detrimental to the host under conditions of infection or microbial colonization, including during* M. tuberculosis* infection, various mechanisms are in place to prevent immunopathology, including those mediated by Foxp3+ regulatory T cells [16, 90, 91, 93] and IL-10 [14, 58–60, 74]. Contrary to what was initially believed, the ability to produce IL-10 has been shown not only in Th2 and Treg [107, 108] cells but also in Th1, Th9, Th17, and CD8+ T cells, especially those that are long-lived [107–119], and the occurrence of these multifunctional populations in the context of IL-10 still needs to be correctly determined in human tuberculosis.

Previous results from our group [33] note an interesting time-dependent effect in the establishment of protective immunity after antimicrobial therapy against* M. tuberculosis*. We demonstrated that the establishment of a Th1 response, characterized by increased production of IFN-*γ* and TNF-*α*, occurs later, as evidenced in patients who had been cured for over 12 months, and is accompanied by increased IL-10 production [33]. This slow development of a Th1 response in human tuberculosis differs from that observed in cutaneous leishmaniasis, a protozoan infection in which the Th1 response is also associated with cure and the Th1 response is induced immediately after treatment [120]. Consequently, our results indicate that although the process of clinical cure progresses with potentiation of Th1 cytokine production (IFN-*γ* and TNF-*α*), the production of higher levels of IL-10 is important for regulating the production of these proinflammatory cytokines [110]. The balance between these regulators and TNF/IFN-*γ* may determine if the immune system can eradicate* M. tuberculosis* with minimum associated damage.

There is a growing body of evidence suggesting that the relationship between IL-10 and Th1 cytokines is not as antagonistic as originally believed, and infectious diseases appear to act in a complementary form [121]. Studies show that, for some infectious diseases, an increase in IL-10 potentially acts to decrease the deleterious effects of inflammation derived from Th1 cytokines without affecting the clearance of infectious agents [121–124], such as* Listeria monocytogenes*,* Trypanosoma cruzi*, and influenza virus [125–127]. Similarly, the protective response to* Toxoplasma gondii* is associated with IFN-*γ*, although in the absence of IL-10, infected animals may die due to extensive early tissue damage [128, 129].* In vitro* approaches suggest that the sources of IL-10 in these infections are Th1 cells and these IL-10-producing Th1 cells still maintain their ability to activate macrophages [121]. CD4+ CD25+ Treg cells are also related to this function, especially in* Leishmania major* infection [90, 117, 118]. Also, during* L. major* infection, although the absence of IL-10 enhanced pathogen clearance, mice displayed a loss of immunity to reinfection, suggesting that IL-10 does limit pathogen clearance but has a key role in the maintenance of effector memory populations via a mechanism that remains unknown [130]. Knockout mice for IL-10 that were infected with* M. tuberculosis* did not show increased IFN-*γ* production [131], although they did show long-term lack of control of inflammatory responses and progression of the disease [61]. Additionally, IL-10 can also enhance inflammatory mediators, especially in an environment that is rich in IFN-*γ* [119].

With regard to the dynamics of IL-10 production in tuberculosis active disease and after clinical cure, the results are conflicting. Some studies indicate that there are higher levels of IL-10 in active tuberculosis [14, 58, 59], but Sahiratmadja and colleagues showed that this increase happens only after clinical cure [74]. Still, Zhang and colleagues showed no change in the levels of IL-10 among individuals with active disease and those who had been treated [60]. Recently, Siawaya and colleagues found that, during antituberculosis therapy, there were no major changes in the levels of IL-10; however, patients who had lower levels of this cytokine in the earlier stages had negative cultures for* M. tuberculosis*, indicating better bacterial clearance in these individuals [132]. Pereira and colleagues found that, in addition to an enhancement in the production of TNF-*α*, patients with systemic manifestations of tuberculosis have increased production of IL-10 [73]. These data suggest that IL-10 can have deleterious effects on the patient during active disease. Gerosa and colleagues have previously shown that there is concomitant production of IFN-*γ* and IL-10 by Th1 lymphocytes as well as by memory T cells in bronchoalveolar lavage fluid obtained from patients with active tuberculosis [133]. If the IL-10 somehow acts as a weapon used by the bacilli to interfere with proper macrophage activation during active infection, participation in controlling exacerbation of the immune response seems to be of vital importance to the infected individual [134].

In recent years, special attention has been given to so-called double-cytokine-producing effector T cells and their role in mediating the immune response in several infectious diseases, especially protagonists of the Th1 response, which is the case with toxoplasmosis, malaria, and leishmaniasis [33, 121, 135–137]. Initially, Th2 cells were the main populations of T cells that produced IL-10 in a sustained manner [138–140]. Later, several groups reported an associated production of IFN-*γ* and IL-10 [121, 141–144] and subsequently IL-17 and IL-10 [124]. These studies have not yet been able to determine if these populations are stable or are only transitory stages, where the production of IL-10 occurs only transiently and returns to its initial profile [145].

Experimental models suggest that the generation of IL-10-producing Th1 cells is due to chronic or repeated antigenic stimulation [146–149] because newly differentiated Th1 cells are apparently unable to secrete IL-10 due to the inaccessibility of the IL-10 gene promoter [139]. In fact, as mentioned earlier, several studies have indicated that the expression of IL-10 in cells that were initially committed to rigid profiles, such as Th1, Th2 or Th17, may reflect a mechanism of self-control after repeated antigenic stimulation where cell survival should be extended. Previous work in experimental models demonstrated that external factors, such as antigenic load and increased presence of IL-12, may direct Th1 cells to expression of IL-10, which returns to baseline upon removal of these stimuli [23, 150]. Moreover, this production is dependent on both the induction of STAT-1, which is classically associated with Th1 and IFN-*γ* production, and STAT-3, particularly via the IL-27-IL-27R axis [146, 147]. In Th17 cells, production is dependent on STAT-3 and IL-27-IL27R axis after induction by IL-6 and IL-21 [148–150]. Studies indicate that the IL-10/STAT-3 axis is essential for the development and function of CD8+ memory T cells [154–156] and expansion of these cells with IL-10 production capacity occurs during chronic infection with* M. tuberculosis* [157].

In addition to the aforementioned signaling pathways, the signaling receptor Notch has been shown to be essential for production of IL-10 by CD4+ T cells, as well as the IFN-*γ*+ and IL-10+ double-producing T cells [158, 159]. These receptors have also been associated with IFN-*γ*, especially via Notch1 and Notch2 during* Leishmania major* infection [160]. Indeed, stimulation of the Notch pathway via DLL-4, a prominent Notch receptor ligand, is described as guiding STAT-3, and consequently, IL-10 [156] and Notch signaling pathway has also been associated with increased survival of T cells [161], data that together could strengthen understanding of the occurrence of these double-producing cells in chronic pathological processes. Current results from our group indicate that a higher percentage of CD4+ cells express Notch1 in patients who are clinically cured of tuberculosis, a condition that is characterized by increased IFN-*γ* and IL-10.

Furthermore, FoxP3− CD4+ memory cells, especially those that express CCR6, are induced by IL-10 and secrete the same cytokines with the purpose of self-regulation [162]. We should also consider that the decrease in IFN-*γ* and TNF-*α* in the supernatants of these cell strains can only be the result of apoptosis of IL-10-producing Th1 cells after their suppression. Several studies have also indicated that antiapoptotic functions of IL-10 are important because blocking this cytokine makes non-Hodgkin's lymphoma cells more susceptible to apoptosis [163] and increases cell apoptosis in the retina [164]. In addition, IL-10 prevents T-cell mediated apoptosis by parainfluenza virus type 3 (PIV3) [165] and EBV [166] and inhibits apoptosis of cardiomyocytes, monocytes, and macrophages [167, 168], especially via the induction of STAT-3 [169].

Over the years, several studies have examined the ability of DNA vaccines based on mycobacteria HSPs, particularly HSPs from* M. tuberculosis* and* M. leprae*, to provide protection against pulmonary tuberculosis. Interestingly, although the results indicate their ability to induce protective immunity against* M. tuberculosis* by enhancing effector populations of CD4 and CD8+ T cells and increasing IFN-*γ* [170–175], they also induce regulatory T cells and IL-10 for potential inhibition of autoimmune manifestations [147–149, 176–178], demonstrating a practical situation of success in controlling infection with* M. tuberculosis* with effector/regulator concurrent mechanisms.

In the context of human infection with* M. tuberculosis*, we understand that the influence of IL-10 on inhibiting apoptosis of T cells is crucial for maintaining the repertoire of cells that are responsible for bacillary control, maintenance of clinical latency, long preservation of lymphocyte life and memory, and therefore the ability to produce those cytokines that are related to protection and preservation of granulomas.

## 4. Conclusions

Based on careful surveys of the literature and published works from our group, we have come to the following conclusions:

- The balance between resistance and susceptibility to infection with* M. tuberculosis* is a very complex mechanism and presents several controversies over the cytokine profiles of T helper cell subpopulations.
- IL-10 appears to have an important role in shaping the repertoire of T helper lymphocytes, irrespective of other cytokines that define their subpopulations.
- Chronic infection in the presence of IL-10 appears to exert a regulatory role and minimize tissue damage, but without compromising the immune mechanisms involved in the control of* M. tuberculosis* and other related microorganisms.

## Figures and Tables

**Table 1:** T helper cell (Th) subtypes and Th-related soluble mediators in human and experimental tuberculosis.

	Species	Putative role in tuberculosis	Reference
*T helper subtype *			
Th1	Mouse and human	↓ the growth and dispersion of *M. tuberculosis *	[7–11]

Th2	Human	↑ in BALF associated with clinical progression	[12]
		↓ Th2 response in active disease	[13, 14]
	Mouse	↑ progression and reactivation of TB	[15]

T regulatory	Human	↑ Treg cells in more severe active disease	[16]
		↑ reactivation of latent TB	[17]
		↑ TB-MDR	[18]
	Mouse	↑ Treg cells in the early days of infection protects from severe disease	[19]

Th17	Human	↓ Th17 cells in active TB	[20, 21]
	Mouse	↑ neutrophil accumulation and tissue damage	[22, 23]
		↑ recruitment of IFN-*γ* producing cells	[24]
		Induce granuloma formation and remain as long-lived memory cells	[25–27]

Th22	Human	↑ in healthy *M. tuberculosis*-exposed individuals	[20]
		↑ in PBMC culture from patients with active TB	[28]
		↑ in pleural fluid from patients with active TB	[29–31]

Th9	Human	↑ Th9 cells in tuberculous pleural effusion	[32]

*T helper-related soluble mediator *			
IFN-*γ*	Human	↑ *Mycobacterium*-specific production after clinical cure	[33]
		↑ in active TB patients at the site of infection	[34–37]
	Mouse	↓ influx of neutrophils and neutrophil-associated tissue damage	[38]
		↑ iNOS in infected macrophages	[39, 40]
	Human and mouse	↑ autophagy	[41]

NO	Mouse	↑ killing and growth inhibiting of virulent *M. tuberculosis *	[39]

TNF-*α*	Human	↑ *Mycobacterium*-specific production after clinical cure	[33, 42–44]
	Mouse and human	Maintenance of the granuloma integrity	[45–48]

IL-4	Human	↑ in the blood and BALF in TB patients with severe forms	[12, 49–53]
	Mouse	↑ progression and reactivation of TB	[15, 54]
		Without any influence in disease susceptibility	[55, 56]
	Mouse and human	↓ autophagic control of intracellular *Mycobacterium tuberculosis *	[57]

IL-10	Human	↑ *Mycobacterium*-specific production after clinical cure	[33]
		↑ in active disease	[14, 58, 59]
		No difference between active-TB and clinically cured individuals	[60]
	Mouse	↓ long-term lack of control of inflammatory responses and progression of the disease	[61]

IL-17	Mouse	Th1 induction following BCG vaccination	[62]

IL-9	Human	↑ in patients with pulmonary TB	[63]
		↓ IFN-*γ* expression by PBMCs in latent TB	[64]

IL-22	Human	↑ in pleural fluid from patients with active TB	[29–31]
	Mouse	IL-22 deficiency does not alter the outcome of infection	[32, 65]
